# A top-down view of the tumor microenvironment: structure, cells and signaling

**DOI:** 10.3389/fcell.2015.00033

**Published:** 2015-05-29

**Authors:** Rahul Bhome, Marc D. Bullock, Hajir A. Al Saihati, Rebecca W. Goh, John N. Primrose, A. Emre Sayan, Alex H. Mirnezami

**Affiliations:** ^1^Cancer Sciences, Faculty of Medicine, University of SouthamptonSouthampton, UK; ^2^University Surgery, University of Southampton, Southampton General HospitalSouthampton, UK; ^3^Department of Experimental Therapeutics, MD Anderson Cancer CenterHouston, TX, USA

**Keywords:** tumor microenvironment, cancer stroma, cancer-associated fibroblast, extracellular matrix, microRNA

## Abstract

It is well established that the tumor microenvironment (TME) contributes to cancer progression. Stromal cells can be divided into mesenchymal, vascular, and immune. Signaling molecules secreted by the tumor corrupts these cells to create “activated” stroma. Equally, the extracellular matrix (ECM) contributes to tumor development and invasion by forming a biologically active scaffold. In this review we describe the key structural, cellular and signaling components of the TME with a perspective on stromal soluble factors and microRNAs (miRNAs).

## Introduction

The relationship between tumor and stroma is symbiotic. Stromal cells are corrupted by malignant epithelium, creating a permissive microenvironment which drives cancer progression (Hill et al., 2005). Unlike cancer cells which transform through a series of genetic mutations, stromal cells are mostly genetically intact (Boehm et al., 1997; Allinen et al., 2004). Targeting this component of the tumor microenvironment (TME) should therefore be considered in cancer therapy. There has been an exponential rise in research into this field (reviewed in Witz, 2009). Table 1 summarizes the important studies.

**Table 1:** **Key findings which have defined the TME**.

**Era**	**Key finding: relevance to TME**	**Reference(s)**
Late 1800s	“Seed and soil” hypothesis: a specific microenvironment is required for tumors to establish at a secondary site.	Paget, 1889
Early 1970s	Tumor angiogenesis factor isolated: birth of angiogenesis.	Folkman et al., 1971
Mid 1970s	Macrophages first identified in TME of solid tumors: characterization of immune TME.	Hersh et al., 1976; Russel et al., 1976
Early 1980s	Tumor cells shown to digest extracellular matrix components: the importance of the extracellular matrix in tumor invasion.	Jones and DeClerck, 1980
Early 1980s	Soluble factors from tumor cells stimulate colony formation of normal cells: the role of transforming growth factors in the TME.	Moses et al., 1981; Nickell et al., 1983
Mid 1980s	Fibroblasts shown to exchange genetic material with HeLa cells *in vitro*: a mechanism for stromal-tumor interaction identified.	Delinassios and Kottaridis, 1984
Mid 1990s	Extracellular matrix induces β-casein gene expression in mammary cells: TME elements can alter gene expression in tumor cells.	Roskelley et al., 1994
Mid 2000s	MicroRNAs are shuttled between cells in extracellular vesicles: novel cell-cell communication in the TME.	Valadi et al., 2007
Mid 2010s	Exosomes from fibroblasts alter breast cancer cell polarity and induce invasion and therapy resistance.	Boelens et al., 2014

*This table chronologically lists key findings and their relevance to our understanding of the TME*.

**Figure 1:**
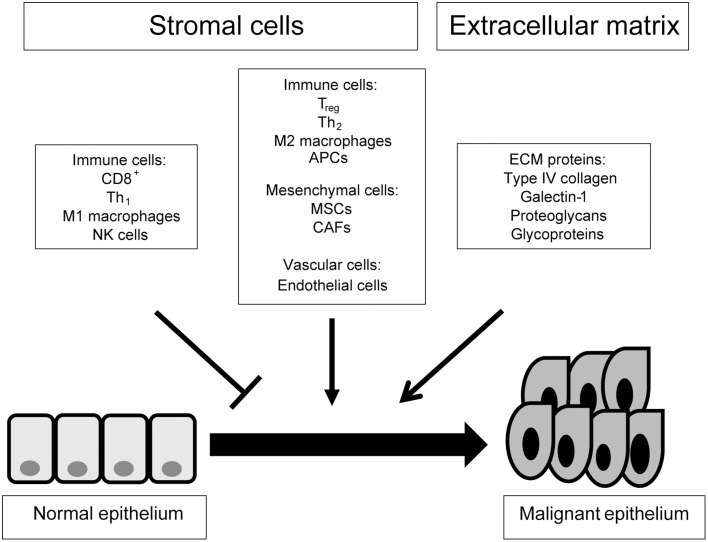
**A top down view of the tumor microenvironment**. This scheme gives an outline of the cellular and acellular components of the tumor microenvironment and their contribution to tumorigenesis. ECM, extracellular matrix; APC, antigen presenting cell; NK, natural killer; T_reg_, regulatory T cell; T_h_, helper T cell; CAF, cancer-associated fibroblast; MSC, mesenchymal stem cell.

There is a difference between TME and stroma that should be defined at the outset. The stroma is a histological unit consisting of peri-tumoral cells within an extracellular scaffold. The TME is a functional ecosystem of tumor and stromal elements that interact through signaling molecules (Figure 1). This review provides a compositional description of TME components which are relevant to cancer progression. Additionally we have listed the signals provided by the stroma that effect cancer cells, including cell-extracellular matrix interactions, soluble factors such as cyto/chemokines and extracellular vesicles such as exosomes.

## Mesenchymal cells

### Cancer-associated fibroblasts

Cancer-associated fibroblasts (CAFs) are the predominant cell type in the stroma, responsible for the structural architecture of the extracellular matrix (ECM; Kalluri and Zeisberg, 2006). They modulate the ECM by expressing several key proteins such as periostin (Kikuchi et al., 2008) and tenascin-C (De Wever et al., 2004). In normal physiology, α-SMA positive fibroblasts (myofibroblasts) have a contractile function to close a wound. In the cancer setting, myofibroblasts remain persistently activated, facilitating cancer progression (Marsh et al., 2013). Desmoplastic tumors characterized by a dense stromal reaction do not always contain myofibroblasts (Chang et al., 2011), suggesting that not all CAFs are myofibroblasts.

It is not clear how CAFs contribute to tumourigenesis but studies have demonstrated neoplastic transformation in their presence (Hayward et al., 2001). Olumi et al. (1999) showed that when human prostate CAFs were co-cultured with normal prostate epithelial cells, they stimulated rapid epithelial growth and altered histology. Moreover, simulation of CAF signaling by Wnt-1-transfected fibroblasts caused morphological transformation in mammary epithelial cells (Jue et al., 1992). Importantly, secretion of soluble factors such as transforming growth factor-β (TGF-β) and hepatocyte growth factor by stromal fibroblasts was shown to induce malignant transformation (Kuperwasser et al., 2004)

In early cancer, the host tissue is remodeled to accommodate the developing tumor. Microscopically this is characterized by compositional changes and stiffening of the ECM (Bonnans et al., 2014). For this to happen, collagen is cross linked with other ECM molecules such as elastin, a process catalyzed by lysyl oxidase (LOX; Erler et al., 2006). CAFs produce LOX and collagen in sufficient quantities to facilitate this process (Levental et al., 2009). Importantly, LOX inhibitors such as beta-aminopropionitrile and magnolol synergistically reduce migration and invasion of MDA231 breast cancer cells (Chen et al., 2012).

CAFs play an important role in angiogenesis. When CAF-secreted fibroblast growth factor-2 (FGF-2) is inhibited, angiogenesis is reduced (Pietras et al., 2008). Furthermore, brivanib (a dual VEGF/ FGF tyrosine kinase inhibitor) effectively blocks angiogenesis in a pancreatic neuroendocrine tumor model (Allen et al., 2011). This is of particular interest because selective inhibition of VEGF alone with bevacizumab leads to drug resistance (Lieu et al., 2013). Targeting stromal and cancer-driven angiogenic signaling in combination may present a more effective treatment option. Importantly, the angiogenic effects of CAFs are not limited to their local environment. For example, fibroblast expression of stromal cell-derived factor-1 (SDF-1/ CXCL12) acts as a systemic chemotactic signal for circulating immature endothelial cells (ECs), leading to breast cancer vascularization and metastasis (Orimo et al., 2005).

There is growing evidence suggesting that CAFs induce invasiveness and metastatic capability of cancer cells. Epithelial-mesenchymal transition (EMT) is a cellular programme that induces cancer cell metastasis (reviewed in Kalluri and Weinberg, 2009). CAFs promote this transition in multiple cancers (Yu et al., 2014; Zhou et al., 2014). Moreover, there is evidence to suggest that CAFs guide metastatic cells to prime the secondary site for colonization (Xu et al., 2010).

### Mesenchymal stem cells

Mesenchymal stem cells (MSCs) are defined by their adherence properties, ability to differentiate into different cell types and surface markers (CD73, CD90, and CD105; Dominici et al., 2006). At least 20% of CAFs originate from MSCs and recruitment is dependent on TGF-β and SDF-1 (Quante et al., 2011). CAFs abundantly express these chemotactic signals. Additionally, cancer cells can induce differentiation of MSCs to CAFs. Indeed, the exposure of human MSCs to conditioned media from MDA231 breast cancer cells stimulated expression of myofibroblast markers such as α-SMA (Mishra et al., 2008).

Weinberg and colleagues marked the importance of MSCs for breast cancer metastasis. Once recruited to the TME, stromal MSCs secrete CCL5 (RANTES) and enhance the metastatic capability of breast cancer cells (Karnoub et al., 2007). Similarly, HS-5 human bone marrow stromal cells increased proliferation, migration, and invasion of Huh7 hepatocellular cancer cells. These functional effects were attenuated by knocking down CCL5 (Bai et al., 2014).

## Immune cells

Immune cells in the TME can have pro- or anti-tumor effects. Tumor progression can be stunted or inhibited by immunosurveillance but established tumors and metastases have the ability to modify the TME in order to escape immunity (Vesely et al., 2011). The immune response produced by M1 macrophages, T helper-1 cells, cytotoxic T cells, antigen presenting cells (APCs), and natural killer (NK) cells supports tumor rejection; whereas, M2 macrophages, regulatory T cells, and T helper-2 cells support tumor progression (Verbeke et al., 2011).

### Macrophages

Macrophages are phagocytic cells that play a critical role in innate and adaptive immunity. They are classified into pro inflammatory M1 and anti-inflammatory M2 subtypes. M1 macrophages take part in immunosurveillance. Through the release of pro-inflammatory cytokines such as interleukin-1 and tumor necrosis factor alpha (TNF-α), they inhibit tumor progression (Noy and Pollard, 2014). Conversely, M2 macrophages release immunosuppressive cytokines such as interleukin-10 and allow tumor progression (Mantovani et al., 2002; Solinas et al., 2009); hence they are called tumor-associated macrophages (TAMs). TAMs have been shown to facilitate tumourigenesis and tumor progression in colonic (Liu et al., 2011a) and renal cell (Daurkin et al., 2011) carcinomas, respectively. The effects of TAMs are not limited to immune modulation. In melanoma, TAMs promote endothelial recruitment and angiogenesis via the release of adrenomedullin (Chen et al., 2011). In another study, TAMs directly enhanced the invasiveness of SKBR3 breast cancer cells by exporting a certain microRNA (miR-223) in extracellular vesicles (Yang et al., 2011).

### T-lymphocytes

CD8+ cytotoxic T cells induce growth arrest, necrosis, and apoptosis in tumor cells by the release of various cytokines including interferon gamma (IFN-γ; Matsushita et al., 2015). The residual protein components of the apoptotic cells are then phagocytosed by APCs and exposed to maturing lymphocytes in lymphoid organs (Chiang et al., 2015). This potentiates tumor suppression. Conversely, regulatory T cells (Tregs) promote immune tolerance by expressing a cytokine profile that attenuates the proliferation of CD8+ cells, inhibits APCs and macrophages and reduces the lytic activity of NK cells (Facciabene et al., 2012). This potentiates tumor progression. Indeed, Tregs have been found in higher numbers in various cancers such as liver (Gao et al., 2007) and breast (Bates et al., 2006). In theory, therapies that increase the proportion of CD8+ cells to Tregs may attenuate tumor progression. For example, lympho-depletion followed by autologous transfusion of tumor-infiltrating CD8+ cells in patients with metastatic melanoma prompted clinical and radiological regression of metastases in 18 of 35 human subjects (Dudley et al., 2005). More recently, the anti-CD25 monoclonal antibody daclizumab has been shown to suppress Tregs and enhance anti-tumor response (Ohmura et al., 2008). In another study, daclizumab depleted CD25-high Tregs and allowed an enhanced IFN-γ-mediated CD4/ CD8+ response in metastatic breast cancer patients (Rech et al., 2012).

There are several classes of CD4+ helper T (Th) cells but Th1 and Th2 are functionally prominent in cancer progression (Knutson and Disis, 2005). Th1 cells are necessary for the activation and persistence of CD8+ cells. Indeed, intravenous injection of antigen-specific Th1 cells induced CD8+ cell-mediated tumor regression in a fibrosarcoma model (Surman et al., 2000). The role of Th2 cells is less clear but in patients with renal carcinoma and melanoma, circulating CD4+ cells display Th2-polarized (IL-5) responses to MAGE-6 epitopes in active disease and Th1-polarized (IFN-γ) responses in remission (Tatsumi et al., 2002). Similarly, CD4+ cells from patients with stage I renal carcinoma showed predominantly Th1-polarized responses to EphA2, whereas CD4+ cells from later stages showed progressively more Th2-polarized responses (Tatsumi et al., 2003). Overall, the presence of Th2 cells marks poor prognosis compared to Th1 cells.

### Antigen-presenting cells

APCs process and display antigens with MHC proteins to naïve T cells. MHC I-expressing cells stimulate CD8+ cells whereas MHC II-expressing cells stimulate CD4+ cells. In general, APCs are classified into professional and non- professional cells. The most important professional APCs are dendritic cells (DCs). Fibroblasts are an example of non-professional APCs which do not constitutively express MHC II but can stimulate T-cells by expressing IFN-γ (Sprent, 1995).

APCs from the TME of rat colonic carcinoma did not stimulate CD8+ cells as well as non-tumor APCs (Chaux et al., 1997). This was attributed to a lack of co-stimulatory factor B7, suggesting that cancer cells make APCs functionally deficient. Human renal and pancreatic cancer cell lines express interleukin-6 and macrophage colony stimulating factor, which alter the differentiation of APCs from CD34+ to CD14+ progenitors. CD14+ cells express little MHC II and cannot evoke a significant immune response, thereby lacking APC function and allowing tumor escape (Menetrier-Caux et al., 1998; Bharadwaj et al., 2007). Furthermore, in the presence of malignant cells, APC progenitors differentiate into immature myeloid-derived suppressors (Almand et al., 2001) and TAMs (Cortez-Retamozo et al., 2012), both of which are immunosuppressive.

### Natural killer cells

NK cells are innate immune cells which are important in halting tumor progression (Eguizabal et al., 2014). NK cells destroy tumor cells in animal models of several human cancers (reviewed in Schiavoni et al., 2013) by detecting cell surface changes such as reduced MHC I (Karre et al., 1986). In an immunocompetent environment, NK cells select out APCs which do not express MHC I sufficiently, thereby maintaining a pool of APCs which are best equipped to present foreign antigens (Moretta, 2002). However, NK-mediated immunity is dampened in the TME by tumor-secreted cytokines such as TGF-β (Wilson et al., 2011). NK cells isolated from triple negative breast cancers exhibited less antibody-mediated cytotoxicity, a phenomenon that can be reversed by addition of the pro-inflammatory IL-2/IL-15 complex (Roberti et al., 2012). In contrast to this, microarray analysis of intra-tumoral NK cells from non-small cell lung cancer patients showed upregulation of pro-cytotoxic genes (NKp44, granzyme-A and -B) compared to extra-tumoral NK cells (Gillard-Bocquet et al., 2013). To explain this, the authors propose that NK cells are activated but functionally exhausted in the tumor setting. Thus the activity, rather than presence, of NK cells suggests good prognosis in cancer patients.

## Vasculature

The stromal vasculature is made up of a capillary network of ECs surrounded by pericytes that provide structural and physiological support. It is well established that hypoxia limits tumor progression and that this drives angiogenesis (Folkman, 1971). The pathophysiology of angiogenesis has been extensively reviewed elsewhere (Otrock et al., 2007).

### Endothelial cells

ECs in the TME are microscopically different to regular ECs. They lack a pericyte covering, have leaky tight junctions and exhibit sprouting (Carmeliet and Jain, 2011). Stromal tissue hypoxia triggers the release of VEGF from pericytes, activating VEGF-2 receptors on adjacent ECs. These ECs become “tip” cells and migrate toward hypoxic tissue that has the highest VEGF concentration. ECs lagging behind the “tip” bind to each other through surface ligand-receptor complexes, slowing their own migration and allowing the chain of ECs to lengthen, or sprout (Gerhardt et al., 2003). At reduced oxygen concentration, the expression of hypoxia inducible transcription factors (HIF) -1 and -2 by ECs is upregulated (Wong et al., 2013). HIF-1 is associated with increased proliferation and migration of ECs (Tang et al., 2004), whereas HIF-2 promotes EC maturation and quiescence (Skuli et al., 2009). Deletion of both HIF-1 and -2 suppresses primary tumor invasion but whereas HIF-1 deletion reduces metastasis, HIF-2 deletion increases it (Branco-Price et al., 2012). The evidence suggests that modulation of HIFs and their downstream effects may well be a therapeutic option in parallel to established anti-VEGF treatments.

## Extracellular matrix

ECM constitutes the cellular scaffold of the TME providing structural support to tumor epithelium and stromal cells alike. ECM is produced by mesenchymal cell types including fibroblasts, chondrocytes, and osteoblasts and consists of various components including collagens, galectins, proteoglycans, and glycoproteins (Denys et al., 2009). ECM has the capacity to both initiate and channel signaling cascades within the TME (Hynes, 2009), and through bidirectional interplay with malignant cells, impact upon tumor progression and metastasis (Murphy et al., 1992; Sadej et al., 2008). Furthermore, its biomechanical properties determine to an extent the dynamics of ECM turnover, thus influencing the ability of malignant cells to invade (Egeblad et al., 2010). Equally, ECM may provide a “cancer stem-cell” niche and is implicated in angiogenesis and inflammation pathways which contribute to a pro-metastatic TME. (Reviewed by Lu et al., 2012). In the following section we will highlight the key components of ECM, their roles during disease progression and their clinical relevance in cancer.

Type IV collagen is the major component of the basement membrane (Mariyama et al., 1992), and possibly the most important protein in the ECM, that separates epithelium from stroma. In desmoplastic tumors such as pancreatic carcinoma, type IV collagen, like other ECM proteins, binds to integrin receptors on cancer cells, promoting their survival (Ohlund et al., 2013). Galectin-1 is a carbohydrate binding protein with several important effects on tumor cells namely: adhesion to the ECM, increased migration and stromal immune suppression (Rabinovich, 2005). Proteoglycans such as heparan sulfate maintain the physical connections between different ECM components (Vlodavsky and Friedmann, 2001). Indeed, salivary gland tumors expressing more heparanase are associated with poorer survival (Ben-Izhak et al., 2006). Glycoproteins such as fibronectin and laminin-1 are ligands for β-integrins, cellular proteins which mediate cell-ECM signaling (Hynes, 2002). ECM expression of fibronectin and laminin-1 correlates with poor prognostic features in breast cancer (Ioachim et al., 2002). In a 3-dimensional breast cancer model, inhibition of fibronectin/α5β 1-integrin binding prompted apoptosis and greater radio-sensitivity (Nam et al., 2010).

The key enzymes regulating ECM turnover are matrix metalloproteinases (MMPs) and tissue inhibitors of metalloproteinases (TIMPs). MMPs are zinc-dependent endopeptidases, capable of degrading almost all ECM proteins. Increased MMP expression is associated with most tumors (McCawley and Matrisian, 2000). Traditionally, it was thought that cancer cells secreted MMPs in order to digest the ECM and permit invasion (Recklies et al., 1980). We now know that MMPs are secreted by both tumor and stromal cells and are important in other aspects of cancer progression such as angiogenesis (Gonzalez-Villasana et al., 2015) and metastasis (Che et al., 2015). TIMPs negatively regulate MMP activity. TIMP-3 is most specific to the ECM (Gu et al., 2014). When breast cancer and ocular melanoma cell lines were transfected with TIMP-3 and injected into nude mice, tumor growth was significantly reduced (Anand-Apte et al., 1996). Methylation of the TIMP-3 gene promoter is the mechanism by which TIMP-3 is inactivated in cancer (Gu et al., 2008). TIMPs are not simply MMP inhibitors. TIMP-3 for example, prevents VEGF from binding to the VEGF-2 receptor, thereby inhibiting angiogenesis (Qi et al., 2003).

## New players in stroma-cancer cell interaction: MicroRNAs

In recent years it has become evident that stroma tumor interaction is not simply composed of paracrine signaling of soluble factors and cell-matrix adhesion. Lipid membrane bound small vesicles, namely exosomes, are secreted from both cancer and stromal cells and influence gene expression of cells in the vicinity (Valadi et al., 2007). These vesicles deliver their RNA and protein cargo and alter gene expression in the recipient cells. Among their cargo miRNAs stand out as major players because they are relatively stable compared to mRNA and proteins and can therefore accumulate to a level that can exert a stable biological effect (Valadi et al., 2007). MiRNAs are non-coding RNAs that are approximately 20 nucleotides long. They undergo enzymatic activation in the cytoplasm, where they bind to the 3' untranslated region of coding mRNAs to prevent protein translation (reviewed in Mirnezami et al., 2009). MiRNAs regulate a variety of cellular processes such as proliferation, differentiation, and apoptosis (Esquela-Kerscher and Slack, 2006). Aberrant miRNAs fail to properly regulate these processes, leading to malignant transformation (Calin et al., 2002). The association between certain miRNA signatures and certain cancers (Grange et al., 2014; Wang et al., 2014), has fueled great interest in miRNAs as diagnostic and prognostic tumor markers. Additionally, stromal exosomes were shown to induce cancer cell behavior with RNA transfer (Boelens et al., 2014). Below we discuss three important stromal miRNAs that can play a role in cancer progression by different mechanisms.

MiR-21 is an oncomir and associated with aggressive colorectal cancer (CRC; Liu et al., 2011b). Importantly, recent articles robustly proved that CRC cells themselves do not express miR-21; it is produced by stromal fibroblasts (Nielsen et al., 2011; Bullock et al., 2013). It is quite likely that stromal mir-21 is transferred to CRC cells by exosomes. MiR-21 directly targets the tumor suppressor protein PDCD4 and protects cancer cells from apoptosis (Asangani et al., 2008). A secondary miR-21 target is reversion-inducing cysteine- rich protein with Kazal motifs (RECK; Gabriely et al., 2008; Reis et al., 2012). RECK is an MMP inhibitor which prevents degradation of the ECM. MiR-21-transfected fibroblasts express less RECK and more MMP-2, permitting greater invasion by CRC cells (Bullock et al., 2013).

Bronisz et al. (2012) defined an axis between the tumor suppressor protein PTEN, miR-320 and the oncogenic transcription factor ETS2. When the PTEN gene was selectively ablated from mouse mammary fibroblasts, miR-320 expression was reduced and ETS2 expression was increased. This led to activation of an oncogenic secretome from stromal fibroblasts, responsible for promoting tumor angiogenesis and invasion. To corroborate this, microarray analysis of 126 human breast carcinomas showed a significant inverse correlation between miR-320 and ETS2. Thus a stromal microRNA can induce a pro-oncogenic/inflammatory secretome to contribute to cancer progression.

There is an important stromal miRNA locus (miR-212/132 family) which regulates normal breast development (Ucar et al., 2010). When miR-212/132 is deleted, MMP-9 increases and collagen deposition around mammary ducts is altered. This corresponds with peri-ductal TGF-β activation, leading to abnormal ductal outgrowths. It will be of great interest to study whether miR-212/132 is de-regulated in breast cancer stroma by altering the ECM and activating TGF-β.

## Conclusion

Taking a systemic approach we have dissected the components of the TME and their relevance to cancer progression. We have described several potential therapeutic targets in the TME besides cancer cells. Perhaps the ubiquity of these targets in normal and malignant tissue is a limiting factor. Nonetheless, a better understanding of the TME will be the key to overcoming this problem.

## Author contributions

AM conceptualized this review. RB and AM decided on the content. RB and AS drafted the work with significant contributions from MB, HS, JP. RG prepared the figure. All authors approve the final version of the manuscript and agree to be accountable for all aspects of the work.

### Conflict of interest statement

The authors declare that the research was conducted in the absence of any commercial or financial relationships that could be construed as a potential conflict of interest.
